# Publisher Correction: The mutational impact of culturing human pluripotent and adult stem cells

**DOI:** 10.1038/s41467-020-17797-y

**Published:** 2020-08-04

**Authors:** Ewart Kuijk, Myrthe Jager, Bastiaan van der Roest, Mauro D. Locati, Arne Van Hoeck, Jerome Korzelius, Roel Janssen, Nicolle Besselink, Sander Boymans, Ruben van Boxtel, Edwin Cuppen

**Affiliations:** 1 Center for Molecular Medicine and Oncode Institute, University Medical Center Utrecht, Universiteitsweg 100, 3584 CG Utrecht, The Netherlands; 2 Leibniz Institute on Aging—Fritz Lipmann Institute, Beutenbergstraße 11, Jena, 07745 Germany; 3 Princess Máxima Center for Pediatric Oncology, Heidelberglaan 25, 3584 CS Utrecht, The Netherlands

**Keywords:** Genomics, Computational biology and bioinformatics, Mutation, Adult stem cells, Pluripotent stem cells

Correction to: *Nature Communications*10.1038/s41467-020-16323-4, published online 19 May 2020.

The original version of this Article contained errors in the Results and Discussion sections, where 8 base substitution types incorrectly read “C > T.” The correct version states “C > A” in place of “C > T.”

The Results also incorrectly represented 3 base substitution types with the symbol “–”. The correct version uses “>” instead of “–”.

This has been corrected in both the PDF and HTML versions of the Article.

