# Supplementary material for: The mutational impact of culturing human pluripotent and adult stem cells
Source: Nat Commun. 2020 May 19;11:2493. doi: 10.1038/s41467-020-16323-4 (PMC7237696; doi:10.1038/s41467-020-16323-4)
Supplement: Supplementary file 2 — Description of Additional Supplementary Files [file 41467_2020_16323_MOESM2_ESM.pdf]

**Description of Additional Supplementary Files**

File Name:Supplementary Data 1
Description: Details on the cultures and genetic variants of all the subclones

File Name:Supplementary Data 2
Description:List of the genes that are affected by single base substitutions
