# Supplementary material for: The mutational impact of culturing human pluripotent and adult stem cells
Source: Nat Commun. 2020 May 19;11:2493. doi: 10.1038/s41467-020-16323-4 (PMC7237696; doi:10.1038/s41467-020-16323-4)
Supplement: Supplementary file 5 — Reporting Summary [file 41467_2020_16323_MOESM5_ESM.pdf]

nature research

Corresponding author(s): Ewart Kuijk
Edwin Cuppen

Last updated by author(s): Feb 24, 2020

Reporting Summary

Nature Research wishes to improve the reproducibility of the work that we publish. This form provides structure for consistency and transparency in reporting. For further information on Nature Research policies, see Authors & Referees and the Editorial Policy Checklist.

Statistics

For all statistical analyses, confirm that the following items are present in the figure legend, table legend, main text, or Methods section.

n/a	Confirmed	
☐	☒	The exact sample size (*n*) for each experimental group/condition, given as a discrete number and unit of measurement
☐	☒	A statement on whether measurements were taken from distinct samples or whether the same sample was measured repeatedly
☐	☒	The statistical test(s) used AND whether they are one- or two-sided<br>*Only common tests should be described solely by name; describe more complex techniques in the Methods section.*
☒	☐	A description of all covariates tested
☒	☐	A description of any assumptions or corrections, such as tests of normality and adjustment for multiple comparisons
☐	☒	A full description of the statistical parameters including central tendency (e.g. means) or other basic estimates (e.g. regression coefficient) AND variation (e.g. standard deviation) or associated estimates of uncertainty (e.g. confidence intervals)
☐	☒	For null hypothesis testing, the test statistic (e.g. *F*, *t*, *r*) with confidence intervals, effect sizes, degrees of freedom and *P* value noted<br>*Give P values as exact values whenever suitable.*
☒	☐	For Bayesian analysis, information on the choice of priors and Markov chain Monte Carlo settings
☒	☐	For hierarchical and complex designs, identification of the appropriate level for tests and full reporting of outcomes
☒	☐	Estimates of effect sizes (e.g. Cohen's *d*, Pearson's *r*), indicating how they were calculated

Our web collection on statistics for biologists contains articles on many of the points above.

Software and code

Policy information about availability of computer code

Data collection	No software was used for data collection
Data analysis	Filtered vcf files and supporting code are available at https://github.com/UMCUGenetics/in_vitro_mutational_load. The following software was used: STAR v.2.4.2a, Sambamba v0.5.8, HTSeq-count 0.6.1p1, DESeq v1.28.0, DESeq2.0 v1.20, GATK, SNVFI (available at https://github.com/UMCUGenetics/SNVFI), SnpEff, MutationalPatterns (https://bioconductor.org/packages/3.6/bioc/html/MutationalPatterns.html), Gene definitions for H19 were retreived with TxDb.Hsapiens.UCSC.hg19.knownGene from BioConductor. Epigenetic status datasets were downloaded from the ENCODE website (https://www.encodeproject.org/) as BED files. Functional consequences of coding mutations were identified using the VariantAnnotation R package from BioConductor.<br>Driver probabilities were modelled with custom code: https://github.com/bastiaanvdroest/DriverProbabilities.

For manuscripts utilizing custom algorithms or software that are central to the research but not yet described in published literature, software must be made available to editors/reviewers. We strongly encourage code deposition in a community repository (e.g. GitHub). See the Nature Research guidelines for submitting code & software for further information.

Data

Policy information about availability of data

All manuscripts must include a data availability statement. This statement should provide the following information, where applicable:

- Accession codes, unique identifiers, or web links for publicly available datasets
- A list of figures that have associated raw data
- A description of any restrictions on data availability

The source sequencing data underlying Figure 1c-e and Figures 2-4
are available at the European Genome-phenome Archive through (http://www.ebi.ac.uk/ega/) under accession numbers EGAS00001002955, EGAS00001000881 and EGAS00001001682. All the other data supporting the findings of this study are available within the article and its supplementary information files and from the

October 2018

corresponding author upon reasonable request. Publicly available data was also used from dbSNP (dbSNP v137.b37) and ENCODE (https://www.encodeproject.org/).

Field-specific reporting

Please select the one below that is the best fit for your research. If you are not sure, read the appropriate sections before making your selection.

☒ Life sciences ☐ Behavioural & social sciences ☐ Ecological, evolutionary & environmental sciences

For a reference copy of the document with all sections, see nature.com/documents/nr-reporting-summary-flat.pdf

Life sciences study design

All studies must disclose on these points even when the disclosure is negative.

Sample size	Sample sizes were determined based on previous sequencing results (Blokzijl et al., Nature, 2016; Jager et al., Nature Protocols, 2019; Kuijk et al., Science Advances, 2019). The degree of variation between the samples of the same cell type justified the sample sizes.
Data exclusions	No data was excluded from the analysis
Replication	Experiments were performed on biological replicates to verify the reproducibility of the experimental findings. In total 18 subclones were sequenced. Clones and subclones were established for one pluripotent embryonic stem cell line under 20% Oxygen (for which 1 subclone was sequenced), two induced pluripotent stem cell lines under 20% oxygen (iPS line 1: 3 subclones, iPS line no2: 1 subclone) one pluripotent embryonic stem cell line under 3% Oxygen, two induced pluripotent stem cell lines under 3% oxygen (1 subclone each), two liver ASC lines, for which two subclones per line were sequenced, and two intestinal ASC lines, for which three subclones per line were sequenced.
Randomization	From bulk cultures, random individual cells were used for clonal expansion
Blinding	Not applicable to our study design, which is an unbiased approach where all the data is analyzed systematically using the same filtering pipelines independent of subjective interpretation.

Reporting for specific materials, systems and methods

We require information from authors about some types of materials, experimental systems and methods used in many studies. Here, indicate whether each material, system or method listed is relevant to your study. If you are not sure if a list item applies to your research, read the appropriate section before selecting a response.

Materials & experimental systems

n/a	Involved in the study	
☒	☐	Antibodies
☐	☒	Eukaryotic cell lines
☒	☐	Palaeontology
☒	☐	Animals and other organisms
☐	☒	Human research participants
☒	☐	Clinical data

Methods

n/a	Involved in the study	
☒	☐	ChIP-seq
☒	☐	Flow cytometry
☒	☐	MRI-based neuroimaging

Eukaryotic cell lines

Policy information about cell lines

Cell line source(s)	Human induced pluripotent stem cells derived from renal epithelial cells from a healthy donor, human embryonic stem cell line H9 (Thomson et al 1998) was a kind gift from Niels Geijsen, human intestinal stem cells and human liver stem cells were previously derived by us as described in Blokzijl et al 2016
Authentication	Whole genome sequencing confirmed the identity of the cell lines
Mycoplasma contamination	Cell lines have been tested and are free of mycoplasma
Commonly misidentified lines (See ICLAC register)	No commonly misidentified cell lines were used in the current study

Human research participants

Policy information about studies involving human research participants

Population characteristics	iPS cells were derived from two healthy anonymous male research participants
Recruitment	Primary cells were derived from the urine of two healthy anonymous male research participants who were not involved in this study and who have provided explicit informed consent for ips cell derivation and genetic characterization
Ethics oversight	Integration-free human induced pluripotent stem cells were established from urinary cells using Sendai virus with ethical approval of the University Medical Center Utrecht (the Netherlands) under study ID NL55260.041.15 15-736/M. Primary cells were collected by centrifugation of the urine of two healthy anonymous male research participants who were not involved in this study and who have provided explicit informed consent for ips cell derivation and genetic characterization

Note that full information on the approval of the study protocol must also be provided in the manuscript.
